# Chronic Pain Management Approaches among Spanish Physiotherapists: Influences, Practices, Barriers, and Challenges

**DOI:** 10.3390/jpm14090903

**Published:** 2024-08-26

**Authors:** Ángeles Díaz-Fernández, Irene Cortés-Pérez, Esteban Obrero-Gaitán, Ana Raquel Ortega-Martínez, María Catalina Osuna-Pérez, Noelia Zagalaz-Anula, Rafael Lomas-Vega

**Affiliations:** 1 Department of Health Sciences, University of Jaen, Campus las Lagunillas s/n, 23071 Jaen, Spain; andiaz@ujaen.es (Á.D.-F.); eobrero@ujaen.es (E.O.-G.); mcosuna@ujaen.es (M.C.O.-P.); nzagalaz@ujaen.es (N.Z.-A.); rlomas@ujaen.es (R.L.-V.); 2 Department of Psychology, University of Jaen, Campus las Lagunillas s/n, 23071 Jaen, Spain; arortega@ujaen.es

**Keywords:** chronic pain, physiotherapy, biopsychosocial, barriers and facilitators

## Abstract

This study evaluated Spanish physiotherapists’ orientations toward biopsychosocial and biomedical approaches in chronic pain management through a cross-sectional survey of 447 registered professionals. Validated questionnaires assessed knowledge, attitudes, and beliefs. Multivariate analysis of covariance (MANCOVA) identified influential factors and ordinal regression determined the frequency of biopsychosocial application. Content analysis of open-ended responses explored barriers to biopsychosocial implementation. Over 50% of physiotherapists favored the biopsychosocial model, influenced by interdisciplinary work settings, advanced pain knowledge, and specific training. Comprehensive pain knowledge significantly impacted both biomedical and biopsychosocial orientations inversely. The biomedical approach was more prevalent among those with lower education levels and less pain knowledge, particularly at the beginning or over 20 years into their careers. Despite the theoretical preference for biopsychosocial among Spanish physiotherapists, practical application was infrequent, with only 9.8% always using it and 40.7% frequently. Self-reported confidence and skills were crucial determinants of biopsychosocial implementation frequency. Significant barriers included inadequate psychological skills (63.6%), coordination challenges (47.6%), time constraints (43.6%), patient misconceptions (34.2%), and systemic issues. These findings align with international research, highlighting the need to bridge the gap between theoretical knowledge and clinical practice. Addressing these challenges through targeted training and systemic reforms is crucial for improving chronic pain management globally.

## 1. Introduction

Chronic pain is a widespread public health problem that affects around 30% of the global population [1]. Recent data from Spain shows an increase in chronic pain prevalence from 18% to 25.9% [2], highlighting the growing challenge within the country. Chronic pain disrupts daily activities, work, mental health, and relationships [3]. Implementing effective pain management practices is crucial for enhancing the quality of life and reducing the impact on individuals and society [4].

The traditional approach to pain management follows a biomedical (BM) framework, which emphasizes physical aspects but avoids psychological and social influences [5,6,7]; this approach is limited in addressing the complex nature of chronic pain. In contrast, the biopsychosocial (BPS) model considers biological, psychological, and social factors in pain management, offering a more holistic approach [8,9,10,11]. Recent studies recommend transitioning toward a BPS approach to better address the emotional and social effects of chronic pain, in addition to physical symptoms. This patient-centered strategy not only aims to enhance the effectiveness of pain management interventions [12,13], but also aligns with the latest scientific research. Clinical research supports the BPS model by recommending incorporating various techniques, including physical therapy with active exercises and increasing functionality, such as graded activity [14] and graded exposure in vivo [9,15]. Patient empowerment [16,17] and pain education [18,19,20,21] are also core components of the BPS approach [22]. In addition, integrating cognitive–behavioral therapies (CBT) [23,24,25,26], like cognitive functional therapy (CFT) [27,28] or acceptance and commitment therapy (ACT) [24,29], focusing on psychological flexibility [30,31], along with effective pain coping strategies [32], mindfulness-based stress reduction [33,34,35], communication techniques [36], goal setting [13], and addressing psychological concerns in patients [34,37] are essential. This integrated approach, recently known as psychologically informed physical therapy (PIPT) [38,39] or psychologically informed practice (PiP) [40,41,42,43], emphasizes a comprehensive strategy that targets the multifaceted nature of pain. Its primary objective is to prevent the transition from acute to chronic pain and minimize pain-related disability by simultaneously combining physical, behavioral, and psychological interventions within physiotherapeutic care.

Throughout this study, we investigated chronic pain in general without focusing on a specific type or location of pain. This broad approach reflects the diverse range of chronic pain conditions encountered by physiotherapists in their daily practice, including low back pain, cervical pain, shoulder pain, and fibromyalgia, among others. Consequently, the primary obstacle in chronic pain management is the significant gap between the theoretical knowledge of the BPS model and its practical application in patient care [31,44,45]. While the BPS model provides a comprehensive framework for addressing the complex dimensions of chronic pain, and although many physiotherapists support this approach, their practices frequently diverge from this model [31] and integrating its multifaceted strategies into daily clinical practice is often challenging [13]. This discrepancy also impacts patient experiences negatively, deviating from recommended management strategies and affecting the alignment of physiotherapists’ practices with clinical guidelines [46,47,48]. Other difficulties in implementing the BPS model include limited time or resources and healthcare system priorities, which can obstruct its implementation [13,44,45,46,47,49]. Specifically, a lack of accurate training for physiotherapists can result in difficulties delivering interventions effectively and following established protocols [46,47,48,50,51]. Extensive research has been conducted on health care providers’ chronic pain management worldwide, examining influencing factors and barriers to implementation [44,45]. However, there is a lack of specific exploration of Spanish physiotherapists, and understanding whether Spain’s healthcare system, physiotherapist training, and chronic pain management practices present unique challenges compared to global studies is crucial. Addressing these issues can help improve physiotherapy practices in Spain and provide valuable insights into the worldwide scientific literature, potentially influencing health policies and physiotherapy education.

Based on these considerations and addressing the critical need to identify gaps in the practical application of the BPS model in Spanish physiotherapists, this study presents the following objectives: (1) to describe Spanish physiotherapists’ attitudes toward BM and BPS approaches in managing chronic pain; (2) to identify determinants of these attitudes; (3) to explore Spanish physiotherapists’ frequency of BPS approach utilization in clinical settings and analyze influential factors; and (4) to investigate obstacles hindering the implementation of the BPS approach in the clinical practice of Spanish physiotherapists. 

## 2. Materials and Methods

### 2.1. Study Design and Participants

**Figure 1:**
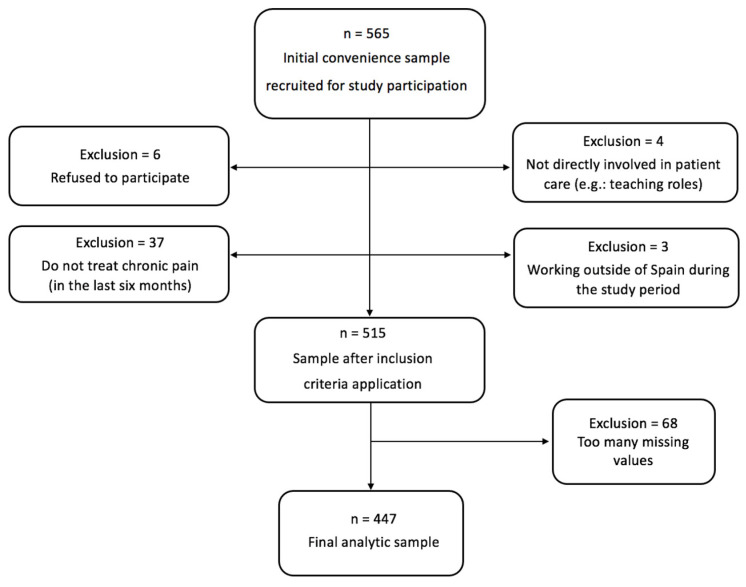
Flowchart of the study.

Following the STROBE guidelines [52], this cross-sectional study was carried out from the end of 2023 and the first months of 2024. It included licensed physiotherapists actively treating chronic pain patients in various healthcare settings in Spain. Six physiotherapists declined to participate without giving reasons. Exclusion criteria comprised non-clinical roles, practicing overseas during the study period, or having not treated chronic pain patients in the last month. A total of *n* = 447 physiotherapists met the eligibility criteria for analysis, as shown in Figure 1.

### 2.2. Ethical Approval and Data Protection

This research followed national and institutional guidelines and the Declaration of Helsinki. University of Jaen’s Ethical Committee approved protocol (ref: SEPT.23/4 PRY). Informed consent was obtained, and data were anonymized and secured to protect participant confidentiality.

### 2.3. Data Collection Procedure

Data were gathered from southern Spain’s physiotherapists through a non-probabilistic intentional sampling of volunteers to efficiently capture a diverse representation of those managing chronic pain across diverse healthcare settings. Participants were invited to participate and recruited from private practices, university health science courses, and public healthcare services. A detailed in-person survey was conducted to ensure authentic responses and reduce bias. It collected sociodemographic, professional, and contextual data and information on chronic pain treatment practices, frequency of BPS approach application, self-perceived skills, and self-confidence levels. An open-ended question was also included to identify barriers to implementing the BPS approach. Participants’ privacy was safeguarded, with researchers having access only to email addresses, and participants were allowed to request clarification at any survey stage. 

### 2.4. Measurements

(a) Sociodemographic, professional, and contextual variables

The survey collected data on aspects potentially affecting physiotherapists’ clinical practices in chronic pain. The variables included were gender, age, work experience, time since graduation, employment type, specific chronic pain training, highest educational level, work setting, and familiarity with implementing evidence-based practice (EBP).

(b) Questionnaires for chronic pain assessment

This study uses four validated and widely employed questionnaires in related research to assess different aspects of chronic pain (knowledge, attitudes, and beliefs) among Spanish physiotherapists.

 
  - Pain attitudes and beliefs scale for physiotherapists (PABS-PT)

Houben et al. [53] examined the original Pain Attitudes and Beliefs Questionnaire for Physiotherapists [54] and created a shorter 19-item version to evaluate physiotherapists’ attitudes and beliefs toward pain management. Physical therapists had to rate statements using a 6-point Likert scale ranging from “totally disagree” to “totally agree”. This instrument includes two subscales: the BM factor, reflecting a traditional, pathology-focused view of pain, and the BPS factor, representing a holistic, patient-centred approach. The Spanish version of the PABS-PT [55] used in this study showed satisfactory psychometric properties.

 
  - Healthcare providers and impairment relationship scale (HC-PAIRS)

Developed by Rainville et al. [56] and revised by Houben et al. [57], the HC-PAIRS assessed healthcare providers’ attitudes and beliefs about pain and impairment in low back pain (LBP). Each statement is rated on a 7-point Likert scale, with higher scores indicating stronger beliefs about avoiding activity and validating disability in LBP. The HC-PAIRS is a unidimensional scale that measures healthcare providers’ BM treatment approach. The validated Spanish version of the HC-PAIRS was used [58], and its psychometric properties were satisfactory.

 
  - Revised neurophysiology pain questionnaire (R-NPQ)

Moseley et al. developed the Neurophysiology of Pain Questionnaire [59] to assess physiotherapists’ understanding of pain neurophysiology. The reviewed Rasch Analysis instrument resulted in the Revised Neurophysiology Pain Questionnaire (R-NPQ [60]). This tool is recognized for its reliability and validity in evaluating pain neurophysiology knowledge, requiring respondents to answer each item with a true, false, or undecided response. Correct answers are assigned 1 point, and the overall score ranges from 0 to 12, with higher scores indicating greater knowledge. The Spanish version of the R-NPQ [61] was used, and its psychometric properties were satisfactory.

 
  - Knowledge and attitudes of pain (KNAP)

The Knowledge and Attitudes of Pain (KNAP) [62] is a 30-item questionnaire for healthcare professionals to assess their knowledge and attitudes toward current pain neuroscience. Responses are rated on a 6-point Likert scale, from “totally disagree” to “totally agree”, with several reverse-scored items. The questionnaire is divided into two domains about the knowledge and treatment approach of chronic pain. Higher KNAP scores, adjusted using Rasch analysis and ranging from 0 to 150, indicate a closer alignment with modern pain neuroscience and recommended treatment guidelines. Our study used the Spanish version of the KNAP (2024, under review), adapted for the Spanish-speaking physiotherapist population with appropriate psychometrics characteristics: Cronbach’s α coefficient was 0.82 for the first domain and 0.70 for the second. The overall internal reliability was Cronbach’s α = 0.86.

(c) Self-reported measures

The survey included three self-reported measures, each with five response categories, to assess (1) the frequency of BPS approach utilization in clinical practice, (2) the physiotherapists’ self-assessment of their skills in implementing BPS treatment strategies, and (3) their confidence level in effectively treating chronic pain cases. These measures aimed to evaluate the extent to which physiotherapists actively apply BPS principles, their perceived expertise in BPS modalities, and their confidence in managing chronic pain within their professional capacities.

(d) Final open-ended question

Physiotherapists were invited to provide detailed insights on the barriers to implementing the BPS approach in managing chronic pain. The question was not mandatory, and they were encouraged to share their experiences and thoughts freely, emphasizing the importance of honesty for a comprehensive understanding of clinical obstacles. The open-ended question was: “In your professional experience, what are the main difficulties encountered when applying the biopsychosocial approach to chronic pain treatment?”.

### 2.5. Assessment of Chronic Pain Treatment Orientations

This study measured physiotherapists’ orientations toward pain management using total scores for the BM and BPS approaches obtained through the survey. The HC-PAIRS score and the BM subscale of the PABS-PT estimated the total BM score. The total BPS score was calculated by combining scores from the PABS-PT-BPS and the treatment subscale of the KNAP questionnaire. To address variations in Likert scales across these instruments, all scores were standardized to establish continuous variables for statistical analysis, ensuring a unified representation of physiotherapists’ scores toward the BM or BPS approach. 

Moreover, to better capture the comprehensive treatment orientations of Spanish physiotherapists toward chronic pain and recognize that optimal management typically involves a concurrent high BPS and low BM orientation, physiotherapists were categorized to reflect distinct perspectives based on this score combination. Following the methodology of a related study [63], five different global treatment attitudes were established to more accurately represent the varying levels of the BM and BPS approach integration in Spanish clinical practice. This categorization into global treatment attitudes serves merely for descriptive purposes. No further statistical analyses were conducted on these categories, as the primary statistical assessments utilized the combined and standardized BM and BPS scores as continuous dependent variables.

### 2.6. Dependent and Independent Variables

(a) Outcome measures

The primary dependent variables in this study were the total BM and BPS scores, which quantify physiotherapists’ orientations toward BM and BPS approaches, respectively. Additionally, an ordinal variable representing the frequency of BPS methodology implementation in clinical practice was included in the survey, categorized on a 5-point Likert scale from “never” to “always”.

(b) Independent variables

The possible influential variables for the treatment orientations in this study include the sociodemographic, professional, and contextual factors, along with the standardized total score for chronic pain knowledge resulting from the R-NPQ and KNAP- pain physiology subscale. Regarding the frequency of BPS approach implementation as the dependent variable, the two self-reported skills and confidence measures were considered potential variables in addition to the sociodemographic and other factors mentioned above.

### 2.7. Sample Size Calculation

The sample size was calculated using G*Power software (version 3.1.9.6; Heinrich-Heine-Universität Düsseldorf, Düsseldorf, Germany), based on a medium effect size (0.25) with a significance level of 0.05 and a power of 95% to ensure statistical robustness. For the MANCOVA analysis, which included 13 degrees of freedom, 16 groups, and one covariate, the required sample size was determined to be 437 participants. This computation was designed to effectively assess fixed effects, main effects, and interactions in chronic pain management research.

### 2.8. Data Analysis

Data analysis was performed using IBM SPSS Statistics 27.0, with a significance level of *p* < 0.05. Normality was assessed with the Kolmogorov–Smirnov test; non-normal continuous data were summarized using medians and IQRs, while categorical variables were described with frequencies and percentages. Missing data were handled by excluding cases with four or more missing data points and imputing the median for isolated instances. Before the principal analysis, Pearson correlations and exploratory factor analysis (EFA) were used to validate the three standardized and combined scores (pain neurophysiology knowledge and BM and BPS total score). Descriptive statistics were used to examine the distribution of total scores for these combined variables. Quartiles were computed for the standardized scores of the total BM and BPS scores to classify physiotherapists into the following five global treatment attitudes: (1) *purely biomedical*, where therapists scored in the highest quartile for BM and the lowest for BPS; (2) *more biomedical*, with BM scores one quartile higher than BPS; (3) *neutral*, where scores for both approaches fell within the same quartile; (4) *more biopsychosocial*, with BPS scores exceeding BM by at least one quartile, and (5) *purely biopsychosocial*, where physiotherapists scored in the highest quartile for BPS and the lowest for BM [63].

Differences across sociodemographic categories were analyzed using *t*-tests and ANOVA with Bonferroni-corrected post hoc tests. Pearson’s correlation assessed multicollinearity (threshold r > 0.80) and linear relationships between variables. Significant influential factors identified in bivariate analyses were included in a multivariate analysis of the covariance model (multifactorial MANCOVA) to evaluate their effects on BM and BPS scores, classifying variables into categorical and ordinal factors or continuous covariates. For initial analyses, ‘Years since graduation’ was used as a continuous variable to explore linear trends, but it was categorized for MANCOVA to highlight differences over years of graduation and simplify the model. Assumptions, such as homogeneity of variance-covariance matrices, were verified. The effect size was assessed using Wilks’ Lambda, with lower values indicating significant effects (values near 1 suggest minimal effects). Model fit was evaluated using F-statistics and *p*-values (*p* < 0.05), and partial eta squared (η^2^) was used to quantify variance contributions by each independent variable, with thresholds set at 0.01 for small, 0.06 for medium, and 0.14 for large effects. Interaction effects were also examined.

Frequencies and percentages were calculated to show the distribution across categories for three self-reported questions. An ordinal regression model predicted the frequency of implementing the BPS approach, with initial analyses including Kruskal–Wallis tests and Spearman’s correlations to examine variable relationships and differences. Significant predictors were integrated into the ordinal regression, assessing model assumptions and multicollinearity (VIF > 5) [64]. Nagelkerke’s R^2^ quantified the explained variance, interpreted using Cohen’s criteria [65] (R^2^ < 0.02 insignificant, 0.02–0.15 small, 0.15–0.35 medium, >0.35 large). The model’s fit was evaluated using the Chi-square statistic, and interaction terms were included to explore variable interdependence.

Two qualitative research experts manually conducted an independent content analysis of the open-ended responses without software assistance. This analysis included systematic identification and categorization of patterns, themes, categories, and their frequencies. Themes were first identified through open coding and then refined and interconnected through axial coding to explore underlying relationships more deeply. To ensure study reliability and analysis integrity, any coding discrepancies were resolved through consensus agreement [66].

## 3. Results

A total of 447 licensed physiotherapists who regularly treat patients with chronic pain completed the evaluation. The sample consisted of 59.9% females, with a median age of 31 years (IQR: 26–37) and a median work experience of 11 years (IQR: 9–18), predominantly in the private sector (51.2%). A significant proportion (almost 80%) reported specific training in chronic pain, with the majority (nearly 30%) receiving between six and ten hours of specific training. Most held a Master’s degree (63.3%) and worked with other physiotherapists (48.5%). More than half were familiar with EBP (52.1%). Initial analysis indicated median scores of 22 points on the PABS-PT-BM factor (IQR: 20–27) and 25 points on the BPS factor (IQR: 22–28). Detailed data are presented in Table 1.

### 3.1. Evaluation of Chronic Pain Treatment Approaches among Spanish Physiotherapists

#### 3.1.1. Validation of the Combined Variables

Significant Pearson correlations confirmed the validity of the standardized combined variables (total chronic pain knowledge, total BM, and total BPS scores), which are now normally distributed. The EFA results further supported consistent factor structure and internal consistency. Detailed validation data are available in Supplementary Table S1 for reference.

#### 3.1.2. Descriptive Analysis of the Three Combined Variable Scores

**Figure 2:**
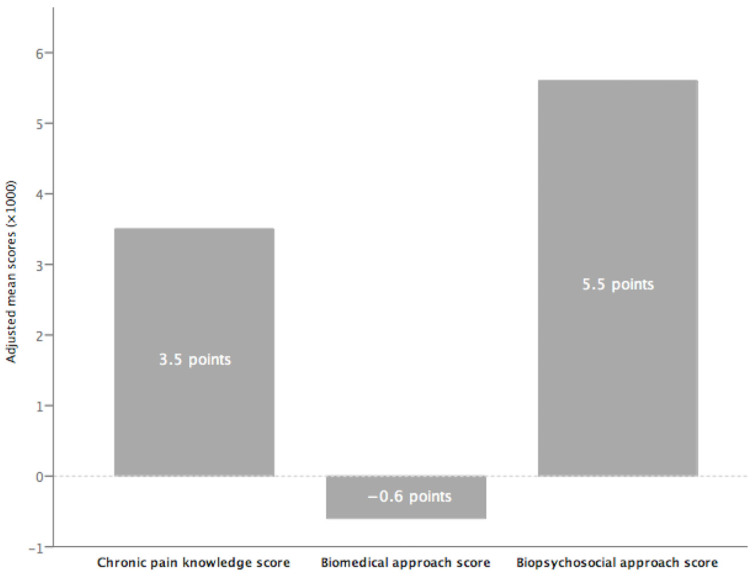
Descriptive analysis of standardized mean scores for chronic pain knowledge, biomedical, and biopsychosocial approaches (*n* = 447).

Figure 2 illustrates the standardized mean scores of Spanish physiotherapists for the three combined variables. Spanish physiotherapists showed a greater inclination to the BPS approach in chronic pain management, as indicated by a significant positive score of +5.5 points. Conversely, the BM orientation is slightly below the neutral point at −0.6, suggesting a minimal deviation from traditional BM models toward a more integrated approach. Regarding chronic pain knowledge, the average score of positive 3.5 points indicates a moderate understanding among participants. Standard deviations were as follows: 1.76 points for chronic pain knowledge, 1.83 points for BM, and 1.92 points for BPS, indicating significant variability in scores among physiotherapists. 

#### 3.1.3. Categorization of Global Treatment Approaches among Spanish Physiotherapists

Physiotherapists were categorized based on their treatment approaches: none adopted a purely biomedical approach; 32.8% demonstrated a more biomedical than biopsychosocial orientation; 17.0% maintained a neutral attitude, integrating both approaches equally; 34.5% favored a more biopsychosocial approach, and 15.7% adhered strictly to biopsychosocial principles. Overall, 50.2% of the physiotherapists used BPS methodologies to treat chronic pain. Further details are provided in Table 2.

### 3.2. Identification Factors Influencing Attitudes toward Biomedical and Biopsychosocial Approaches

Correlation analysis indicated significant positive correlations between age, work experience time, and years since graduation with the BM approach. Additionally, the chronic pain knowledge score showed a strong positive correlation with the BPS approach (r = 0.70; *p* < 0.01) and a strong negative correlation with the BM orientation (r = −0.62; *p* < 0.01). In the bivariate analysis, specific chronic pain training, highest educational level, and work setting had a significant influence on both BM and BPS treatment orientation scores (*p*-values ranging from <0.01 to <0.05), although inversely. Physiotherapists who completed more than 15 h of training in chronic pain tended to adopt the BPS approach. Those with Bachelor’s degrees showed a higher inclination for BM approaches, while individuals with PhDs or pursuing PhD studies were more inclined toward BPS methodologies. Solo practitioners favored the BM model, whereas physiotherapists in interdisciplinary teamwork favored BPS strategies. Table 3 shows bivariate analysis results and correlations for both treatment approaches. Further details are provided in Supplementary Tables S2 and S3.

### 3.3. Results of the MANCOVA Analysis

The correlational analysis identified age, years since graduation, and work experience as three significant continuous independent variables, but the strong correlation among them (r > 0.9) raised concerns about multicollinearity. Therefore, only the variable ’years since graduation’, now categorized into four levels, was chosen for inclusion in the MANCOVA analysis, given its higher Pearson correlation coefficient with both BM and BPS variables than the other related factors. The MANCOVA results, detailed in Table 4, demonstrate that the chronic pain knowledge score deeply impacted both BM and BPS scores, with a Wilks’ lambda of 0.616 and a strong effect size (global partial η^2^ = 0.412). It significantly influenced BM (partial η^2^ = 0.221) and BPS (partial η^2^ = 0.326) orientations. The regression beta coefficients for this variable were for BM (beta): −0.482, t = −11.083, *p* < 0.001; BPS (beta): 0.574, t = 14.460, *p* < 0.001, highlighting its considerable influence.

Details of beta coefficients for all variables are provided in Supplementary Table S4. Interdisciplinary teamwork also significantly influenced treatment orientations (Wilks’ lambda = 0.910), showing lower inclinations for the BM approach and higher for the BPS approach compared to other work settings; post hoc analyses confirmed this different impact on both orientations. Similarly, the number of years since graduation significantly affected treatment orientations. Notably, physiotherapists with over 20 years of experience predominantly favored the BM approach. However, those with less than five years of experience also significantly preferred BM, suggesting an interesting pattern where both very experienced and relatively new professionals lean toward traditional methods. Meanwhile, those with PhDs or pursuing PhD studies showed a stronger inclination toward the BPS approach. Specific training in chronic pain also played a critical role, with those lacking training tending to favor BM attitudes (Wilks’ lambda = 0.967). The MANCOVA analysis indicated robust explanatory power for both BM and BPS orientations, with adjusted R^2^ values of 0.46 and 0.55, respectively, demonstrating significant connections between the identified factors and physiotherapists’ treatment orientations. No significant interactions between independent variables were observed, simplifying the interpretation of direct effects.

### 3.4. Self-Reported Outcomes on BPS Implementation Frequency, Skills, and Confidence

**Figure 3:**
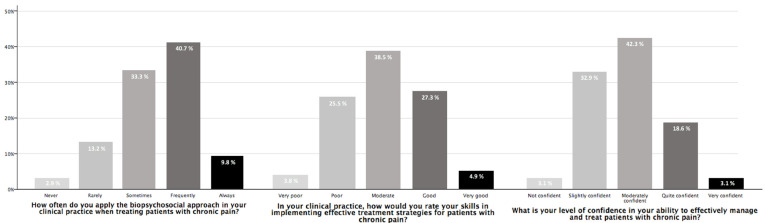
Distribution of self-reported frequency of application, skills, and confidence levels in the BPS approach among Spanish physiotherapists for chronic pain management (*n* = 447).

The analysis of self-reported data is illustrated in Figure 3. A notable 40.7% of physiotherapists reported ‘frequently’ utilizing the BPS approach, with ‘sometimes’ also being the second option response (33.3%). However, it is essential to note that only 9.8% of participants consistently apply the BPS approach ‘always’ in their practice. Additionally, a significant percentage expressed low confidence and skills levels, with ‘moderately confident’ being the most reported confidence level (42.3%) and ‘moderate’ skills (38.5%) being the predominant response in the self-assessed capabilities.

### 3.5. Predicting the Frequency of BPS Approach Implementation

The ordinal regression analysis (Table 5) revealed significant predictors for BPS approach frequency among Spanish physiotherapists: self-reported skills and confidence, knowledge of chronic pain score, and specific training in chronic pain. Remarkably, the highest educational level and work setting, while significant in bivariate analyses, did not predict the frequency in the final regression model. An interaction between self-reported skills and confidence correlated with increased BPS use frequency, enhancing the model’s explanatory power (Nagelkerke’s R^2^ improved from 0.71 to 0.74). The Chi-square test (χ^2^ = 532.449, *p* < 0.001) validated the enhanced model’s fit with the interaction term, confirming analysis robustness and reliability without multicollinearity.

### 3.6. Self-Reported Barriers to the Implementation of the BPS Approach

**Figure 4:**
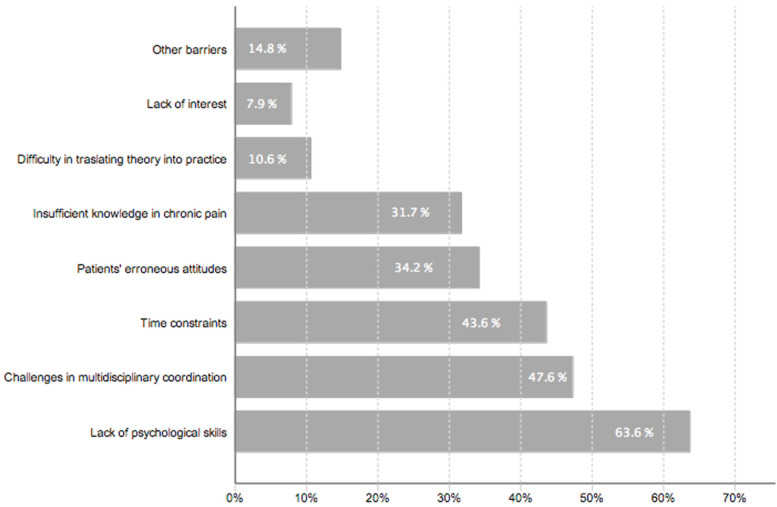
Self-reported barriers among Spanish physiotherapists (*n* = 435) in implementing the biopsychosocial approach in chronic pain management. Percentage of respondents identifying each barrier (derived from content analysis of open-ended responses).

Responses from 435 physiotherapists highlighted numerous barriers to integrating the BPS approach into chronic pain management. A significant 97.3% of participants reported encountering barriers, with over half facing three or more challenges. The primary barrier, reported by 63.6%, was inadequate psychological skills, particularly in effective communication and intervention techniques, and lack of familiarity with patient coping strategies. Close to half (47.6%) cited concerns with multidisciplinary collaboration, including institutional and logistical impediments, role ambiguity, and conflicting team objectives. Time constraints were significant for 43.6% of physiotherapists, pressured by patient volume and insufficient time for holistic care. Erroneous patient attitudes were barriers for 34.2%, with pharmacological or passive treatment preferences. Deficiencies in chronic pain management knowledge were reported by 31.7% of respondents. Other barriers, such as systemic healthcare limitations and inadequate facilities for BPS interventions, were noted by 14.8%. Furthermore, 10.6% of the physiotherapists reported difficulties applying their knowledge about chronic pain and the BPS treatment approach in clinical practice, indicating a gap between educational content and clinical demands. A minority (7.9%) recognized the BPS approach’s value but focused on other pathologies. Table 6 summarizes the barriers with the subthemes identified, and Figure 4 provides a graphical representation. For quotes and a detailed analysis, see Supplementary Table S5.

## 4. Discussion

The main objectives of this study were to investigate Spanish physiotherapists’ orientations toward BM and BPS approaches in chronic pain management, identify influential factors for these orientations, and reveal barriers to implementing the recommended BPS approach. This research underscores the importance of aligning physiotherapists’ practices with contemporary chronic pain management principles, bridging the gap between theoretical knowledge and its clinical application in Spain.

The potential influential variables for this study were determined following a scientific literature review of chronic pain management and physiotherapy [44,45,67,68,69,70,71,72,73,74]. Although most of this literature is on chronic LBP, our study extends these findings to chronic pain in any location, as chronic pain’s characteristics apply beyond specific tissue damage. Variables such as age, years since graduation, clinical experience, type of employment, work setting, knowledge of chronic pain, adherence to EBP, and highest educational level were selected. The variable for ‘speciality’ was excluded from our study due to the lack of formal physiotherapy specializations in Spain, which results in varying individual training and clinical backgrounds among professionals in the country.

Our study’s main finding aligns with the global shift in physiotherapy toward the BPS model over the traditional BM approach. Specifically, 52% of Spanish physiotherapists strongly preferred the BPS model, with a +5.5 points score on the standardized BPS scale. This preference is consistent with international studies advocating holistic pain management [63,68,71,72]. Although some Spanish studies highlight a predominant use of the BM approach among healthcare professionals [75,76], our results support the trend towards BPS methodologies in chronic LBP treatment [77]. However, the enduring presence of BM attitudes underscores the complexity of fully transitioning to BPS practices. 

Our sample of physiotherapists exhibited a moderate understanding of chronic pain, which was lower than expected. The MANCOVA analysis identified high chronic pain knowledge scores as the most influential variable, significantly increasing the inclination toward the BPS approach while reducing the BM approach inclination, as shown by its significant effect size. This result emphasizes the crucial role of adequate pain education in advancing integrated treatment strategies. This finding aligns with Mikamo and Takasaki’s results [74], where pain neurophysiology knowledge was the only significant determinant favoring a BPS-oriented approach among Japanese physical therapists.

Interdisciplinary teamwork was the second decisive factor influencing BPS treatment orientations, with significantly higher BPS and lower BM scores than in other work settings. This outcome highlights the effectiveness of cohesive teams with shared goals and integrated strategies. A scoping review by van Dijk et al. [45] emphasizes the importance of social structures like supervision and management in facilitating BPS adoption. Additionally, Petit et al. [72] found that closer collaboration within LBP care networks correlates with stronger BPS beliefs among physiotherapists, confirming the value of unified teamwork over isolated efforts.

In our research and the study by Christe et al. [73], the age variable was omitted due to multicollinearity with ‘years since graduation’. This influential factor is notable as studies like Ferreira et al. [71] and Benny and Evans [68] indicate that less experienced physiotherapists tend to favor the BM model, aligning with our findings. However, Christe et al. [73] found lesser experience associated with positive attitudes toward back pain, possibly due to different measurement tools. Previous studies often used ‘years since graduation’ as a continuous variable, potentially masking specific professional differences. Our categorization revealed significant trends across experience levels. To address these findings, incorporating BPS principles into both undergraduate and continuing education for physiotherapists is recommended, ensuring early and continuous exposure to BPS concepts.

In our study, physiotherapists with PhDs or pursuing PhD studies favored the BPS approach, suggesting advanced education encourages a more holistic understanding of pain management. This result aligns with Ferreira et al. [71], who observed a similar trend among Brazilian physiotherapists, where those with only an undergraduate degree were more likely to adhere to the BM model than those with a doctorate. However, not all studies validate this observation. However, Benny and Evans [68] found no significant differences based on education level, likely due to a small sample size of PhD physiotherapists, limiting statistical power. Similarly, Alshehri et al. [69] and Mikamo and Takasaki [74] reported no significant influence of postgraduate degrees on BM vs. BPS management. These discrepancies may stem from variations in participants’ demographics, professional backgrounds, and educational systems across countries. Additionally, while the PABS-PT questionnaire was used in these studies, our broader methodological approach, incorporating multiple standardized scores from validated questionnaires, might explain the observed differences.

In our investigation, the key traditional factors showed no significant effects on the treatment orientations of Spanish physiotherapists. Employment type, for instance, did not align with specific treatment models, contradicting Benny and Evans’ findings [68] that public sector workers favored the BPS model. However, we noted a trend supporting the BPS approach in interdisciplinary work settings, which is more predominant in public health centers in Spain, suggesting the work environment may be more influential than the sector itself. Additionally, no gender-based differences in treatment orientation were detected, diverging from previous studies. Additionally, no gender-based differences in treatment orientation were detected, diverging from earlier studies [68,69,71], indicating a female tendency for the BM model. This discrepancy might stem from cultural, methodological, or gender equality variations within our study context. Equally trained and aware physiotherapists regarding the BPS model might have also neutralized expected differences. Regarding EBP, only 52% of participants reported familiarity with it, but familiarity did not correlate with treatment orientation, highlighting a breach in EBP integration that warrants educational and professional development. Unlike studies that did not precisely measure the influence of EBP, supporting Mikamo and Takasaki’s perspective [74], our results suggest that pro-EBP attitudes, research experience, and a supportive environment for research could substantially shape treatment orientations. This finding underscores the need for further research to comprehensively explore how these elements influence the adoption of BPS approaches among physiotherapists.

On the other hand, our self-reported measures indicate that while over 40% of Spanish physiotherapists frequently use the BPS approach in chronic pain management, only about 10% consistently apply it in all cases. Despite regular involvement with chronic pain, these physiotherapists report moderate levels of skills and confidence. This gap highlights significant barriers to fully applying their expertise. The ordinal regression analysis identified chronic pain knowledge, specific training, self-reported skills, and confidence as significant predictors of BPS utilization. The interaction between skills and confidence, crucial elements of professional self-efficacy, notably enhanced BPS application frequency, underscoring the need for targeted training and support. Recent literature, particularly comprehensive reviews of international research [45,78], emphasizes the influence of skills, confidence, and knowledge on physiotherapists’ adoption of the BPS approach in chronic pain management. Additionally, in a systematic review by Ng et al. [44], diverse healthcare professionals identified these factors as critical influences in adopting the BPS model alongside other barriers. Both review findings and our data from Spanish physiotherapists reveal a significant interrelation between skills, confidence, and knowledge, with deficiencies often overlapping and hindering effective BPS implementation.

To better understand the factors affecting physiotherapists’ confidence and perceived competencies, our study used an open-ended question within a qualitative framework, allowing participants to express challenges faced in clinical settings. Nearly all surveyed physiotherapists (97.3%) reported difficulties applying holistic treatment models, primarily due to a lack of psychological skills. About two-thirds identified deficiencies in communication techniques and patient coping strategies, highlighting the need for training programs integrating psychological methods [13,45,47,49,51,78,79]. Additionally, substantial institutional and logistical barriers hinder effective multidisciplinary collaboration among Spanish physiotherapists. Systemic issues such as funding constraints and inadequate facilities further exacerbate these challenges, limiting the resources available for comprehensive, holistic care. Confusion over professional roles, particularly between physiotherapists and psychologists, along with conflicting team objectives, complicates the integration of cohesive pain management strategies. Time constraints often result from high patient loads, limiting the time available for comprehensive care and diminishing the depth and effectiveness of BPS interventions. These findings align with international research, consistently reporting similar organizational and time-related challenges in implementing BPS approaches [46,49,78]. Resistance to the BPS model from patients, noted by about 35% of physiotherapists, emphasizes the need for better patient education to support more holistic treatment approaches. Patients often prefer quick, less involved treatments, like basic pharmacological or even surgical interventions, due to misconceptions about chronic pain, indicating a critical gap in patient understanding that must be addressed [44,45,49]. Furthermore, nearly one-third of Spanish physiotherapists report a lack of up-to-date chronic pain management knowledge as a barrier. This gap, frequently highlighted in international [13,38,44,45,68,71,74,80,81] and national studies [77], reflects the necessity of continuous professional development and integration of the latest research and techniques at all levels of physiotherapy education. Notably, more than 10% of therapists report a gap between theoretical knowledge and practical application, suggesting the need for training in real-world clinical environments. Innovative approaches such as increased patient interaction, role-playing, and clinical simulations are proposed to bridge this theory-practice divide and enhance practical skills for implementing the BPS model effectively [45,51]. Lastly and interestingly, a small fraction (7.9%) recognizes the BPS model’s value but focuses on other clinical areas, perhaps due to less satisfactory outcomes or the complexity of cases. 

This study faces several limitations, such as its cross-sectional design and convenience sampling, which may restrict our ability to infer causality and ensure representativeness across the broader population of Spanish physiotherapists, potentially affecting the generalizability of the results. Nevertheless, the sample includes licensed physiotherapists whose demographic and professional profiles closely align with those of the overall Spanish physiotherapy population, according to 2022 data from the National Statistics Institute [82]. This alignment enhances the study’s representativeness, further validated by its concordance with recent national surveys on low back pain attitudes across different Spanish regions [77]. Despite using a convenience sample, the large size of 447 participants exceeds the pre-calculated requirement for statistical power, partially mitigating representativeness concerns. While integrating variables from various chronic pain and physiotherapy research questionnaires was validated through Pearson correlations and EFA, the interpretation of combined scales may not apply universally, necessitating external validation. Additionally, reliance on self-reported data introduces a risk of social desirability bias, potentially skewing responses toward social expectations rather than reflecting genuine personal beliefs or behaviors.

The study’s strength lies in its innovative approach of integrating validated scores from widely used questionnaires that measure similar constructs, enhancing measurement robustness. This integration was statistically validated through EFA and Pearson correlations, ensuring the combined scales accurately represent the intended constructs. Moreover, this research is the only study in Spain specifically addressing all these aspects of chronic pain management among physiotherapists, filling a significant gap in the existing literature. It provides crucial insights for developing targeted educational programs and policy-making, enhancing the study’s practical impact and relevance for future interventions in chronic pain management.

Further research should explore Spanish physiotherapists’ experiences, attitudes, and challenges with the BPS approach, building on this study’s themes. Mixed-methods research could deepen understanding of adopting the BPS model in chronic pain management. Additionally, other individual differences among physiotherapists, such as other personal beliefs, past experiences with chronic pain patients, intrinsic motivation, and personal experiences with chronic pain, either personally or through close individuals, may also impact the adoption of BPS approaches. Exploring these personal factors can provide deeper insights into the variability in chronic pain management practices. For instance, physiotherapists with positive outcomes with BPS approaches may be more inclined to continue using these methods. In contrast, those who have faced challenges or negative outcomes might revert to more traditional BM approaches. Understanding these individual differences is crucial for developing tailored interventions that address specific barriers and reinforce the benefits of the BPS model. Moreover, evaluating the impact of educational interventions on chronic pain knowledge and psychological skills training is crucial, particularly for enhancing physiotherapists’ confidence. Future studies should use evidence-based methods like role-plays and clinical simulations to measure clinical competencies accurately. Additionally, assessing chronic pain patients’ perceptions of treatments in Spain is necessary to see if improved training and skills enhance patient care outcomes.

This study highlights the need for comprehensive training in modern pain management and psychological techniques for Spanish physiotherapists in terms of clinical implications. Improving these educational aspects will increase confidence and proficiency in the BPS approach. Integrating this training into university curricula and fostering interdisciplinary teams with cohesive goals will enhance patient-centered care in Spain. Additionally, healthcare reforms supporting these educational and collaborative initiatives are crucial for advancing comprehensive pain management strategies within the Spanish healthcare system.

## 5. Conclusions

This study reveals a strong preference among Spanish physiotherapists for the BPS approach in chronic pain management, influenced by chronic pain knowledge, interdisciplinary settings, and educational level. High chronic pain knowledge decreases BM adherence while promoting BPS. Early and late career stages tend to use traditional methods, and a lack of specific training is linked to greater BM reliance. Self-reported skills and confidence, pain neuroscience knowledge and chronic pain training influence the implementation of the BPS approach. Barriers include insufficient psychological skills, multidisciplinary coordination challenges, time constraints, patient resistance to non-medical treatments, and lack of comprehensive pain management knowledge.

Spanish physiotherapists face challenges in holistic chronic pain management similar to those of international trends. Addressing these challenges requires comprehensive training programs, interprofessional collaboration, and updated pain management strategies. Enhancing physiotherapists’ skills and confidence and promoting teamwork are crucial for applying the BPS model effectively. These efforts aim to align theoretical knowledge with clinical practice, improving patient care standards for chronic pain management in Spain and potentially worldwide.

## Figures and Tables

**Table 1:** Sociodemographic profile and questionnaire outcomes of the study participants (*n* = 447).

Variable	Cases (%)	Median (IQR)	Range
Gender			
Female	268 (59.9)		
Male	179 (40.1)		
Age (years)		31 (26, 37)	
Work experience (years)		11 (9, 18)	
Years since graduation (years)		12 (10, 19)	
Type of employment			
Public sector (Hospital)	70 (15.7)		
Public sector (Health centre)	126 (28.2)		
Private sector employment	229 (51.2)		
Self-employed private sector	22 (4.9)		
Specific training in chronic pain			
None	93 (20.8)		
Less than or equal to 5 h	132 (29.5)		
Between 6–10 h	89 (19.9)		
Between 11–15 h	42 (9.4)		
More than 15 h	91 (20.4)		
Highest educational level			
Bachelor’s degree	137 (30.6)		
Master’s degree	283 (63.3)		
PhD student or PhD	27 (6.1)		
Work setting			
Primarily solo practice	108 (24.2)		
Working with other PTs	217 (48.5)		
Multidisciplinary collaboration	66 (14.8)		
Interdisciplinary teamwork	56 (12.5)		
Familiarity with implementing EBP			
Yes	233 (52.1)		
No	214 (47.9)		
RNPQ		7 (5, 9)	(0–12)
HC-PAIRS		58 (53, 75)	(15–105)
PABS-PT			
BM Factor		22 (20, 27)	(8–48)
BPS Factor		25 (22, 28)	(5–30)
KNAP			
KNAP-Pain physiology		64 (56, 86)	(0–84.36) *
KNAP-Treatment		42 (36, 49)	(0–65.36) *

IQR: interquartile range; PTs: physiotherapists; EBP: evidence-based practice; R-NPQ: Revised Neurophysiology Pain Questionnaire; HC-PAIRS: Health Care Providers’ Pain and Impairment Relationship Scale; PABS-PT: Pain Attitudes and Beliefs Scale for Physiotherapists; BM (biomedical factor); BPS (biopsychosocial factor); KNAP: knowledge and attitudes of pain questionnaire; *: Score transformation based on the Rasch method following the authors’ recommendations.

**Table 2:** Profile of Spanish physiotherapists’ five different global treatment approaches (*n* = 447).

	Number of Physiotherapists (*n*)	Percentage (%)	Summary of Categories
Purely biomedical approach	0	–	Biomedical: 32.8%
More biomedical approach	147	32.8	
Neutral approach	76	17.0	Neutral: 17.0%
More biopsychosocial approach	154	34.5	Biopsychosocial: 50.2%
Purely biopsychosocial approach	70	15.7	

Combining quartile scores for the BM and BPS approaches determined the global treatment approach. The “*Summary of categories*” column groups data into three main categories for simplified interpretation.

**Table 3:** Bivariate analysis and correlations of significant factors associated with biomedical and biopsychosocial approaches in chronic pain management.

		BM Approach					BPS Approach				
		Bivariate Analysis			Correlations		Bivariate Analysis			Correlations	
Independent Variables	Reference Category	Mean ± SD	F	*p*-Value	r Pearson	*p*-Value	Mean ± SD	F	*p*-Value	r Pearson	*p*-Value
Work setting: Primarily solo practice *	Interdisciplinary teamwork	0.56 ± 1.60	48.668	0.001	-	-	−0.69 ± 1.59	52.345	0.001	-	-
Work setting: Working with other PTs *		0.09 ± 1.68			-	-	−0.02 ± 1.67			-	-
Work setting: Multidisciplinary collaboration *		0.76 ± 1.57			-	-	−0.77 ± 1.64			-	-
Highest educational level: Bachelor degree *	PhD student or PhD	0.43 ± 1.64	31.529	<0.05	-	-	−0.34 ± 1.70	43.968	0.001	-	-
Highest educational level: Master degree *		−0.40 ± 1.72			-	-	0.38 ± 1.81			-	-
Specific training in CP: No specific training in CP *	More than 15 h	2.78 ± 1.72	24.850	0.001	-	-	−0.72 ± 1.63	16.770	<0.05	-	-
Years since graduation	N/A	-	-	-	0.25	<0.01	-	-	-	−0.14	<0.05
Age (years)	N/A				0.19	<0.01				−0.13	<0.05
Work experience (years)	N/A				0.22	<0.01				−0.14	<0.05
CP knowledge score (standardized)	N/A	-	-	-	−0.62	<0.01	-	-	-	0.70	<0.01

SD: standard deviation; CP: chronic pain; PTs: physiotherapists. *: Dummy variable. N/A: Not applicable.

**Table 4:** Multivariate analysis of covariance (MANCOVA) results and post hoc comparisons for dependent variables (total biomedical and biopsychosocial scores) across the independent variables. *n* = 447. The table includes the beta coefficient for the continuous covariate. No significant interactions between independent variables were found.

Independent Variable		Wilks’ Lambda	F-Value	*p*-Value	df	Global Partial η²	Partial η² by Dependent Variable		Beta Coefficient		t	*p*-Value	Adjusted R^2^	Post-Hoc Comparisons	
							Total BM	Total BPS							
Chronic pain knowledge score		0.616	106.468	<0.001	104, 343	0.412	0.221 *	0.326 *	BM	−0.482	−11.083	<0.001	-	N/A	
									BPS	0.574	14.460	<0.001			
Work setting		0.910	6.971	<0.001	3, 343	0.046	0.064 *	0.038 *	-		-	-	-	Interdisciplinary teamwork < other categories (BM)*	Interdisciplinary teamwork > other categories (BPS )*
Years since graduation		0.926	5.621	<0.001	3, 343	0.038	0.045 *	0.012	-		-	-	-	More of 20 years > all other categories (BM)* Less than 5 years > 6–10 years, 11–20 years (BM)*	
Highest educational level		0.951	5.513	<0.001	2, 343	0.027	0.008	0.038 *	-		-	-	-	PhD student or PhD > other categories (BPS)*	
Specific training in chronic pain		0.967	4.692	0.03	4, 343	0.023	0.016 *	0.008	-		-	-	-	No training > other categories (BM)*	
Overall model	Total BM score	-	5.289	<0.001	104, 343	0.516	-	-	-		-	-	0.46	N/A	
	Total BPS score	-	5.657	<0.001	104, 343	0.622	-	-	-		-	-	0.55		

BM: biomedical; BPS: biopsychosocial; df: degrees of freedom; N/A: not applicable; *: *p*-value < 0.05.

**Table 5:** Bivariate analysis, correlations, and ordinal regression model to predict the frequency of biopsychosocial approach application in chronic pain management, incorporating interaction terms. This table presents the adjusted model, including the significant interaction between self-reported skills and confidence.

Variables	Bivariate Analysis		Correlations		Ordinal Regression Model					
					95% CI for OR					
	H	*p*-Value	Rho Spearman	*p*-Value	Reference Category	B (SE)	*p*-Value	Odds Ratio	Lower Bound	Upper Bound
Self-reported skills	261.968	<0.001	-	-	Very good	2.467 (1.61)	<0.001	11.785	7.893	17.595
Self-reported confidence	165.917	<0.001	-	-	Very confident	0.828 (0.58)	<0.001	2.290	1.648	3.181
CP knowledge score (standardized)	-	-	0.45	<0.001	-	0.290 (0.06)	<0.001	1.336	1.172	1.524
Specific training in CP	39.364	<0.001	-	-	More than 15 h	0.279 (0.83)	0.03	1.321	1.028	1.699
Highest education level	34.074	<0.001	-	-	PhD student or PhD	NS	NS	NS	NS	NS
Work setting	20.286	<0.001	-	-	Interdisciplinary teamwork	NS	NS	NS	NS	NS
**Model fit statistics**									**Fit index**	***p*-value**
Nagelkerke’s R^2^	-	-	-	-	-	-	-	-	0.71	-
Chi square-statistic	-	-	-	-	-	-	-	-	487.844	<0.001
**Adjusted model with interactions**										
Self-reported skills	261.968	<0.001	-	-	Very good	3.028 (1.45)	<0.001	20.652	8.440	50.533
Self-reported confidence	165.917	<0.001	-	-	Very confident	1.430 (0.43)	0.002	4.178	1.679	10.397
CP knowledge score (standardized)	-	-	0.45	<0.001	-	0.289 (0.07)	<0.001	1.335	1.170	1.522
Specific training in CP	39.364	<0.001	-	-	More than 15 h	0.285 (0.81)	0.024	1.330	1.038	1.704
Skills × Confidence interaction	-	-	-	-	-	0.135 (1.23)	0.041	1.119	1.018	1.679
**Model fit statistics with interactions**									**Fit index**	***p*-value**
Nagelkerke’s R^2^	-	-	-	-	-	-	-	-	0.74	-
Chi square-statistic	-	-	-	-	.	-	-	-	532.449	<0.001

CP: chronic pain; SE: standard error; CI: confidence interval; OR: odds ratio; NS: not statistically significant. The reference category is specified for each categorical independent variable.

**Table 6:** Prevalence, themes, and subthemes of barriers to implementing the biopsychosocial approach in chronic pain management identified by Spanish physiotherapists through content analysis of the open-ended question (*n* = 435).

Barrier identified	Frequency (%)	Subthemes Identified
Lack of psychological skills	63.6	Deficient training in effective communication techniques. Insecurity in implementing psychological interventions. Unfamiliarity with patient coping strategies.
Challenges in multidisciplinary coordination	47.6	Institutional barriers to interprofessional collaboration. Lack of understanding of each professional’s role in pain management. Logistical difficulties in organizing multidisciplinary meetings. Divergence in objectives among professionals.
Time constraints	43.6	Pressure to manage high patient volumes. Insufficient session time dedicated to patient education. Prioritization of quick, localized treatment interventions over comprehensive approaches.
Patients’ erroneous attitudes	34.2	Resistance to accepting the BPS model due to preconceptions. Preference for medical (medication) or surgical interventions. Challenges in patient behaviour change and self-care adoption.
Insufficient knowledge of chronic pain management	31.7	Need for specialized training. Limited access to current educational resources. Difficulty in staying updated with current research.
Other barriers	14.8	Healthcare system limitations include funding or policies not favouring the BPS approach. Lack of suitable spaces for conducting BPS interventions.
Difficulty in translating theory into practice	10.6	Discrepancies between received education and real clinical demands. Lack of practical examples during training. Uncertainty in applying theoretical concepts.
Lack of interest	7.9	A general acceptance of the BPS approach’s value, shifting the focus towards other pathologies.

BPS: Biopsychosocial.

## Data Availability

Data used to support the findings of this study are available from the corresponding author upon request.

## Supplementary Materials

The following supporting information can be downloaded at: https://www.mdpi.com/article/10.3390/jpm14090903/s1, Table S1: Statistical validation of combined scores for chronic pain knowledge and treatment approach (*n* = 447). Table S2: Differences in biomedical and biopsychosocial treatment approaches considering categorical or ordinal sociodemographic, professional and contextual factors. (*n* = 447). Table S3: Correlation analysis of treatment approaches with continuous sociodemographic factors and chronic pain knowledge score. Table S4: Detailed beta coefficients for influential factors in chronic pain management approaches among Spanish physiotherapists. *n* = 447. PTs: Physiotherapists. Table S5: Summary of self-reported barriers to implementing the BPS approach in chronic pain management and their frequencies, including representative quotes for each barrier. (*n* = 435).
