# Supplementary material for: Chronic Pain Management Approaches among Spanish Physiotherapists: Influences, Practices, Barriers, and Challenges
Source: J Pers Med. 2024 Aug 26;14(9):903. doi: 10.3390/jpm14090903 (PMC11433413; doi:10.3390/jpm14090903)
Supplement: Supplementary file 1 [file jpm-14-00903-s001.zip › TableS1_Correlations and AFEs_standardized scores.pdf]

Combined variable	Standardized scores	Correlations (r Pearson)	KMO	Bartlett's test of sphericity (*p*-value)	EFA (Cronbach's alpha)	Total variance explained (%)
Total CP knowledge score	R-NPQ + KNAP Pain physiology	0.55**	0.55	p < 0.001	0.71	77.76
Total BM score	PABS-PT-BM + HC-PAIRS	0.61**	0.55	p < 0.001	0.81	83.71
Total BPS score	PABS-PT-BPS + KNAP Treatment	0.67**	0.55	p < 0.001	0.80	83.33

Table S1: Statistical validation of combined scores for chronic pain knowledge and treatment approach (n = 447).

KMO: Kaiser-Meyer-Olkin, measure of sampling adequacy; EFA: Exploratory Factor Analysis; CP: Chronic Pain; BM: Biomedical; BPS: Biopsychosocial; R-NPQ: Revised Neurophysiology of Pain Questionnaire; KNAP-Pain physiology: Knowledge and Attitudes of Pain-Pain Physiology subscale; PABS-PT-BM: Pain Attitudes and Beliefs Questionnaire for Physiotherapists Biomedical Factor; HC-PAIRS: Health Care Providers' Pain and Impairment Relationship Scale; PABST-PT-BPS: Pain Attitudes and Beliefs Questionnaire for Physiotherapists Biopsychosocial Factor; KNAP-Treatment: Knowledge and Attitudes of Pain-Treatment subscale; **: p-value < 0.001.
