# Supplementary material for: Chronic Pain Management Approaches among Spanish Physiotherapists: Influences, Practices, Barriers, and Challenges
Source: J Pers Med. 2024 Aug 26;14(9):903. doi: 10.3390/jpm14090903 (PMC11433413; doi:10.3390/jpm14090903)
Supplement: Supplementary file 1 [file jpm-14-00903-s001.zip › TableS2_Análisis bivariado_diferencias ANOVA_ BM y BPS.pdf]

Independent variable (categorical or ordinal)	Dependent variable (standardized)			
	Biomedical approach	*t* or *F*	Biopsychosocial approach	*t* or *F*
**Gender**				
Female	- 0.07 ± 1.81	0.102	- 0.14 ± 1.76	0.932
Male	0.01 ± 1.86		0.23 ± 1.90	
**Type of employment**				
Public sector (Hospital)	0.25 ± 2.16	0.762	0.31 ± 1.76	1.630
Public sector (Health centre)	0.17 ± 1.86		- 0.26 ± 1.89	
Private sector employment	- 0.11 ± 1.74		0.38 ± 1.81	
Private sector self-employment	0.19 ± 1.32		0.11 ± 1.82	
**Specific training in chronic pain (hours)**				
None	2.78 ± 1.72	24.850**	- 0.72 ± 1.63	16.770*
Less than or equal to 5	0.09 ± 1.55		- 0.01 ± 1.72	
Between 6-10	- 0.66 ± 1.81		0.49 ± 1.91	
Between 11-15	- 1.40 ± 2.45		0.66 ± 1.96	
More than 15	- 2.02 ± 1.10		1.67 ± 0.53	
**Highest educational level**				
Bachelor's degree	0.43 ± 1.64	31.529*	- 0.34 ± 1.70	43.968**
Master's degree	- 0.40 ± 1.72		0.38 ± 1.81	
PhD student or PhD	- 1.52 ± 1.77		2.14 ± 1.33	
**Work setting**				
Primarily solo practice	0.56 ± 1.60	48.668**	- 0.69 ± 1.59	52.345**
Working with other PTs	0.09 ± 1.68		- 0.02 ± 1.67	
Multidisciplinary collaboration	0.76 ± 1.57		- 0.77 ± 1.64	
Interdisciplinary teamwork	- 2.61 ± 1.26		2.31 ± 0.76	
**Familiarity with implementing EBP**				
Yes	- 0.14 ± 1.76	- 1.688	0.01± 1.80	0.001
No	0.15 ± 1.90		- 0.01 ± 2.16	

Table S2: Differences in biomedical and biopsychosocial treatment approaches considering categorical or ordinal sociodemographic, professional and contextual factors. (n = 447). *EBP*: Evidence-based practice. *: p-value < 0.05; **: p-value < 0.001.
