# Supplementary material for: Chronic Pain Management Approaches among Spanish Physiotherapists: Influences, Practices, Barriers, and Challenges
Source: J Pers Med. 2024 Aug 26;14(9):903. doi: 10.3390/jpm14090903 (PMC11433413; doi:10.3390/jpm14090903)
Supplement: Supplementary file 1 [file jpm-14-00903-s001.zip › TableS3_Correlations continous independent variables_BM and BPS.pdf]

Independent variables (continuous)	Treatment approaches (standardized)	
	Biomedical (r Pearson)	Biopsychosocial (r Pearson)
Age (years)	0.19**	− 0.13**
Work experience (years)	0.22**	− 0.13**
Years since graduation (years)	0.25**	− 0.14*
Chronic pain knowledge score (standardized)	− 0.62**	0.70**

Table S3: Correlation analysis of treatment approaches with continuous sociodemographic factors and chronic pain knowledge score. *: p-value < 0.05; **: p-value < 0.01.
