# Supplementary material for: Chronic Pain Management Approaches among Spanish Physiotherapists: Influences, Practices, Barriers, and Challenges
Source: J Pers Med. 2024 Aug 26;14(9):903. doi: 10.3390/jpm14090903 (PMC11433413; doi:10.3390/jpm14090903)
Supplement: Supplementary file 1 [file jpm-14-00903-s001.zip › TableS4_Beta coefficients all variables.pdf]

| | Reference category | Pain management approach | Beta coefficient | SD | t | p-value | Confidence interval (95%) | | Partial Eta Squared |
|---|---|---|---|---|---|---|---|---|---|
| | | | | | | | Lower Bound | Upper Bound | |
| Chronic pain knowledge score | N/A | BPS | 0.574 | 0.40 | 14.460 | < 0.001 | 0.496 | 0.652 | 0.326 |
| | | BM | − 0.482 | 0.93 | − 11.083 | < 0.001 | − 0.597 | − 0.286 | 0.221 |
| Work setting: | Interdisciplinary teamwork | | | | | | | | |
| Primarily solo practice | - | BPS | 0.236 | 0.61 | 1.874 | 0.002 | − 0.036 | 1.522 | 0.008 |
| | | BM | 0.389 | 0.29 | 1.333 | 0.003 | 0.961 | 1.184 | 0.003 |
| Working with other PTs | - | BPS | 0.510 | 0.37 | 1.983 | 0.102 | 0.481 | 0.584 | 0.012 |
| | | BM | − 0.182 | 0.33 | − 2.083 | 0.036 | − 0.021 | − 0.015 | 0.016 |
| Multidisciplinary collaboration | - | BPS | 1.527 | 0.42 | 3.636 | < 0.001 | 0.701 | 2.352 | 0.030 |
| | | BM | − 1.020 | 0.26 | − 3.939 | < 0.001 | − 1.532 | − 0.508 | 0.034 |
| Highest educational level: | PhD student or PhD | | | | | | | | |
| Bachelor degree | - | BPS | 0.907 | 0.43 | 3.543 | 0.006 | 0.871 | 0.953 | 0.029 |
| | | BM | − 0.733 | 0.36 | − 1.735 | 0.048 | − 0.778 | − 0.702 | 0.007 |
| Master degree | - | BPS | 1.025 | 0.27 | 3.901 | < 0.001 | 1.014 | 1.058 | 0.033 |
| | | BM | − 0.389 | 0.40 | − 1.333 | 0.076 | − 0.431 | − 0.352 | 0.003 |
| Specific training in chronic pain: | No training | | | | | | | | |
| Less than or equal to 5 hours | - | BPS | − 0.177 | 0.41 | − 2.763 | 0.504 | − 0.227 | − 0.145 | 0.020 |
| | | BM | 0.432 | 0.32 | 1.976 | < 0.001 | 0.407 | 0.468 | 0.011 |
| Between 6-10 hours | - | BPS | 1.043 | 0.38 | 2.087 | 0.305 | 1.019 | 1.088 | 0.016 |
| | | BM | − 1.379 | 0.45 | − 1.539 | < 0.001 | − 1.425 | − 1.341 | 0.006 |
| Between 11-15 hours | - | BPS | 0.964 | 0.28 | 2.285 | 0.041 | 0.940 | 0.991 | 0.017 |
| | | BM | − 1.063 | 0.37 | − 3.245 | 0.059 | − 1.105 | − 1.032 | 0.028 |
| More than 15 hours | - | BPS | 1.020 | 0.42 | 3.936 | < 0.001 | 0.982 | 1.063 | 0.033 |
| | | BM | − 2.027 | 0.34 | − 3.532 | < 0.001 | − 2.068 | − 2.001 | 0.029 |
| Years since graduation: | More than 20 years | | | | | | | | |
| Less than or equal to 5 years | - | BPS | − 0.165 | 0.23 | − 2.743 | 0.006 | − 0.247 | − 0.193 | 0.019 |
| | | BM | 1.389 | 0.43 | 2.351 | < 0.001 | 1.351 | 1.438 | 0.018 |
| Between 6-10 years | - | BPS | 0.374 | 0.37 | 3.148 | 0.052 | 0.331 | 0.417 | 0.026 |
| | | BM | − 1.739 | 0.46 | − 4.026 | 0.077 | − 1.788 | − 1.702 | 0.036 |
| Between 11-20 years | - | BPS | 0.626 | 0.26 | 2.852 | 0.186 | 0.593 | 0.657 | 0.021 |
| | | BM | − 1.675 | 0.49 | − 3.645 | < 0.001 | − 1.725 | − 1.631 | 0.030 |

**Table S4:** Detailed beta coefficients for influential factors in chronic pain management approaches among Spanish physiotherapists. n = 447. PTs: Physiotherapists.
