# Supplementary material for: Chronic Pain Management Approaches among Spanish Physiotherapists: Influences, Practices, Barriers, and Challenges
Source: J Pers Med. 2024 Aug 26;14(9):903. doi: 10.3390/jpm14090903 (PMC11433413; doi:10.3390/jpm14090903)
Supplement: Supplementary file 1 [file jpm-14-00903-s001.zip › TableS5_Citas exactas fisioterapeutas_barreras BPS.pdf]

Barrier identified	Frequency	Examples of quotes from physiotherapist respondents
Lack of interest	7.9 %	▪ 'I find it hard to stay motivated to apply the BPS approach when immediate results are not seen.'<br>▪ 'Sometimes I find it difficult to stay updated with the BPS approach, simply because I have other priorities that seem more urgent in my daily practice.'<br>▪ 'While I recognize the importance of the BPS approach for chronic pain, currently, my focus is more drawn towards other pathologies that require my immediate attention.'
Difficulty in translating theory into practice	10.6 %	▪ 'Understanding the BPS model is one thing, but applying it effectively in real-world scenarios is another challenge altogether.'<br>▪ 'Understanding the BPS model conceptually is not difficult, but the challenge lies in effectively applying its principles in daily patient interactions.'<br>▪ 'The BPS makes sense in theory, but there's a substantial gap between the training I received and my patients' real needs and expectations, which makes its practical application more complex than it seems.'
Other barriers	14.8 %	▪ 'We struggle with administrative tasks that take away from our patient care time, making it harder to implement comprehensive approaches.'<br>▪ 'Without support from my colleagues, it's challenging to consistently apply the BPS model in my practice.'<br>▪ 'The limited funding and restrictive policies of our healthcare system make it difficult to fully integrate the BPS approach, as resources are often allocated elsewhere.'
Insufficient knowledge of chronic pain management	31.7 %	▪ 'I feel like I don't have enough training in the BPS approach to use it with my patients effectively.'<br>▪ 'Sometimes I've had to seek help to understand what's happening with my patients, what's going on with chronic pain, and I still do.'<br>▪ 'I often find myself in need of more specialized training to keep up with the latest developments in chronic pain management and effectively implement the BPS approach.'
Patients' erroneous attitudes	34.2 %	▪ 'Many of my patients expect quick fixes and don't see the value in the psychological or social aspects of their treatment."<br>▪ 'Patients often have only one goal in mind, and that's for me to take away their pain, and they don't understand (or don't want to understand) any other type of outcome besides that.'<br>▪ 'Often, my patients prefer a pill or a procedure over engaging in the more comprehensive BPS strategies that involve lifestyle changes and active participation.'<br>▪ 'I find a significant barrier in guiding patients towards behaviour changes and self-care practices; they frequently rely on passive treatments rather than taking an active role in their recovery.'

Barrier identified	Frequency	Examples of quotes from physiotherapist respondents
Time constraints	43.6 %	▪ 'Finding the time to incorporate BPS strategies into sessions is difficult with back-to-back appointments.'<br>▪ 'I would love to be able to dedicate the necessary time to provide pain education to my patients, but unfortunately, I don't have it."<br>▪ 'The high volume of patients I need to see daily makes it nearly impossible to deliver the personalized, in-depth BPS approach that I believe in.'<br>▪ 'The current structure of clinic appointments favours quick, symptomatic relief over the time-consuming process of holistic BPS interventions.'
Challenges in multidisciplinary coordination	47.6 %	▪ 'Coordinating multidisciplinary care becomes difficult when there are few joint meetings and patient appointments are scheduled on different days, resulting in a lack of knowledge about each other's contributions and strategies. This fragmented approach hampers our ability to provide a unified approach to managing chronic pain.<br>▪ 'One of the primary challenges I encounter is that every health professional appears to work independently, as though they are on their own individual islands, lacking common objectives. This frequently gives the impression that we are moving towards divergent aims, leading to complications in developing a unified treatment strategy for our patients experiencing chronic pain.'<br>▪ 'Even though I'm part of a multidisciplinary team, I often notice that the doctor, psychologist, and I are pursuing different objectives without a coordinated common strategy, which compromises the effectiveness of the treatment.'
Lack of psychological skills	63.6 %	▪ 'I realize I need more skills in psychological interventions to embrace the BPS approach fully.'<br>▪ 'In clinical practice, I often lack the sufficient ability to motivate patients to move or to try changing their mindset, beyond repeatedly explaining the neurobiology of pain to them.'<br>▪ 'There's a certain apprehension I feel when applying psychological strategies, as my training in this area is quite limited.'<br>▪ 'Often, I feel powerless during a patient's pain flare-up, struggling to explain effectively—and in a way they can understand—the importance of continuing to move and stay active. I'm not sure how to manage these situations.'

Table S5: Summary of self-reported barriers to implementing the BPS approach in chronic pain management and their frequencies, including representative quotes for each barrier. (n = 435).
